# Evaluation of Interface Shear Strength Coefficient of Alternative Geogrid Made from Para Rubber Sheet

**DOI:** 10.3390/polym15071707

**Published:** 2023-03-29

**Authors:** Anubud Liangsunthonsit, Pakkapon Jaroonrat, Jiratchaya Ayawanna, Weerawut Naebpetch, Salisa Chaiyaput

**Affiliations:** 1 Department of Civil Engineering, School of Engineering, King Mongkut’s Institute of Technology Ladkrabang, Bangkok 10520, Thailand; 64601164@kmitl.ac.th (A.L.); 64601106@kmitl.ac.th (P.J.); 2 School of Ceramic Engineering, Institute of Engineering, Suranaree University of Technology, Nakhon Ratchasima 30000, Thailand; 3 Center of Rubber Technology for Community, Faculty of Engineering, Thaksin University, Phatthalung 93110, Thailand

**Keywords:** geogrid, interface shear strength, para rubber, polymer, rubber compound, RSS

## Abstract

In this work, elastic natural rubber compound sheet (RCS) and ribbed smoked sheet grade 3 (RSS) were studied as alternative replacements for polymer geogrid for soil reinforcement. In order to investigate the reinforcing effectiveness in three distinct environments using the interface shear strength coefficient (*R*_in_) by the large-scale direct shear test, the RSS and RCS geogrids were installed independently in sand, lateritic soil, and clay. Using either an RSS geogrid or RCS geogrid, the average *R*_in_ is progressively smaller in reinforced sand, lateritic soil, and clay, respectively. Higher tensile strength of reinforced materials using the RCS geogrid than those using the RSS geogrid is encouraged by the better elastic characteristics of the RCS geogrid. Thus, utilizing the RCS geogrid-reinforced materials can better increase the shear strength of coarse-grained soil such as sand and gravel.

## 1. Introduction

Geosynthetic material is used to provide one or more functions including reinforcement, separation, energy absorption, etc. A geogrid is one type of geosynthetic [1], which is used for the reinforcement function to increase soil strength. Normally, a geogrid is installed within the soil structure or between the soil layers. The use of geogrids for soil reinforcement is very distinctive for the reinforcement of embankments and road construction. Geogrids have three functions, which are the tension membrane effect, the improvement of bearing capacity, and lateral restraining capability [2].

Many researchers have studied the function of geogrids in relation to the improvement of any engineering properties of soil after installation. Bergado et al. (1993) [3] have studied the interaction between reinforcement grids and clay by using a large-scale direct shear test. With geogrid reinforcement, the shear strength of clay increased correspondingly in the study of Nagrale et al. (2010) [4]. Likewise, Hufenus et al. (2006) [5] have reported that geogrids effectively enhanced the California bearing ratio (CBR) of unpaved roads, which were constructed on the weak subgrade.

The reinforcement efficiency was performed through the interface shear strength coefficient. The interface shear strength coefficient is the ratio of the shear strengths of the soil with geogrid reinforcement and the soil without geogrid reinforcement (soil only). The effect of geogrid properties on soil reinforcement has been studied by many researchers [6,7]. According to Raja and Shukla (2020) [8], the interface shear strength coefficient is a crucial aspect to consider when geosynthetic reinforcement is being used since it provides the shearing resistance at the interface between soil and geosynthetics. According to Palmeira and Milligan (1989) [9], the angle between the geogrid and shear direction had an impact on the variance in interface shear strength coefficients of geogrids. Moreover, Sakleshpur et al. (2019) [10] indicated that the aperture size, aperture area, maximum tensile strength, and junction strength of the geogrid influenced the interface shear strength coefficient of soil reinforcement.

One factor that impacts the stability of the soil is its shear strength. Shear strength or shear stress determines most failure patterns in soil structure. When shear stress exceeds the capacity of the soil, soil failure occurs. As a result, a variety of techniques are used to increase the shear strength of the soil.

In recent years, natural alternative materials, which are generally classified as limited life, have been applied to increase the applications in geotechnical engineering work [11,12,13,14]. Natural reinforcing materials with limited life are only required to perform over the short-term time line in geotechnical engineering applications. According to Sarsby (2007) [15], limited-life geosynthetics were used for temporary embankment roads over soft-ground construction. The stability of the embankment improved with time owing to a decrease in pore pressures in the soil foundation and promoting the shear strength of the soil foundation [16].

Para rubber is a well-known agricultural product in Thailand. Recently, the productivity of para rubber has exceeded the demand of the market. Hence the price of para rubber has decreased continuously. To solve this problem, the natural para rubber or ribbed smoked sheet (RSS) was studied to be used for the function of geogrid reinforcement [17,18]. The RSS was applied to increase the CBR of low-CBR lateritic soil. Unlike ordinary synthetic geogrids, which are made of thermoplastic polymer materials such as polyester, polypropylene, or polyethylene, the RSS geogrids are composed of natural rubber, a thermoset material with good chemical resistance, high creep resistance, and high thermal stability [19].

Therefore, the aim of this study is to intensively study the soil-reinforcement efficiency of using a para rubber reinforcement, which was the RSS and the RCS. The interface shear strength coefficient was determined by using the large-scale direct shear test under the various types of soil, which were clay, lateritic soil, and sand.

## 2. Material Properties

### 2.1. Soil Properties

**Figure 1:**
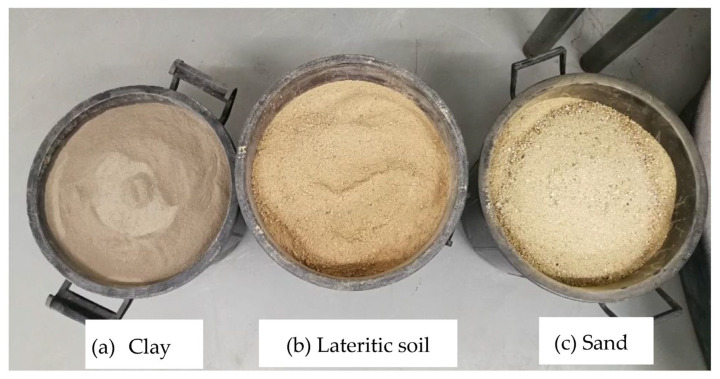
Soil samples: (**a**) clay; (**b**) lateritic soil; (**c**) sand.

In this study, soil samples were sand, clay, and lateritic soil. Before testing, the soil samples were dried by heating in the oven at the temperature of 150 °C for 48 h. After drying by heating, dried clay was pounded and passed through sieve no 200. The soil samples were separately stored in dry containers for each type of soil. The containers were sealed to prevent the infiltration of moisture from the outside of the container into samples. All soil samples were tested for determining their engineering properties, which included particle size analysis and Atterberg’s limits. The dry soil samples in the containers are shown in Figure 1.

**Figure 2:**
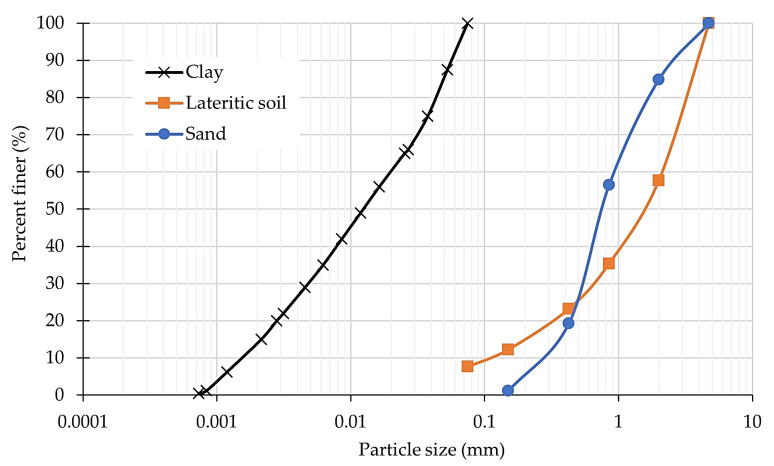
The particle size distribution of clay, lateritic soil, and sand.

The clay used in this study was collected from Samut Prakan province, Thailand (Figure 1a). The clay sample was natural clay at 4 m to 7 m depth from the ground surface. The clay sample was classified as inorganic silt (MH) according to the unified soil classification system (ASTM D2487-17) [20] with a liquid limit, plastic limit, and plastic index of 70.4%, 39.74%, and 30.66%, respectively (ASTM D4318) [21]. The distribution curve of clay particles is shown in Figure 2.

The lateritic soil was collected from the soil plant of the KG construction company, located in the eastern area of Saraburi province, Thailand (Figure 1b). The lateritic-soil sample was classified as clayey sand (SC) (ASTM D2487-17) [20], and the diameter of the largest particle was 2 mm. The particle diameter at 10% (*D*_10_), the particle diameter at 30% (*D*_30_), the particle diameter at 50% (*D*_50_), and the particle diameter at 60% (*D*_60_) were 0.1, 0.69, 1.70, and 2.05 mm, respectively. The minimum particle diameter (*D*_min_) was 0.075. The uniformity coefficient (*C*_u_) and the coefficient of gradation (*C*_c_) were 20.5 and 2.32, respectively. The distribution curve of lateritic soil’s particles is shown in Figure 2.

The sand was collected from the Lopburi river in the middle plain of Lopburi province, Thailand (Figure 1c). According to the unified soil classification system (ASTM D2487-17) [20], the sand sample was classified as poor-grade sand (SP). The *D*_10_, *D*_30_, *D*_50_, and *D*_60_ were 0.29, 0.53, 0.74, and 0.91 mm, respectively. The minimum particle diameter (*D*_min_) was 0.15. The *C*_u_ and the *C*_c_ were 3.14 and 1.06, respectively. The particle distribution curve of sand is shown in Figure 2.

### 2.2. Reinforcement Material

**Figure 3:**
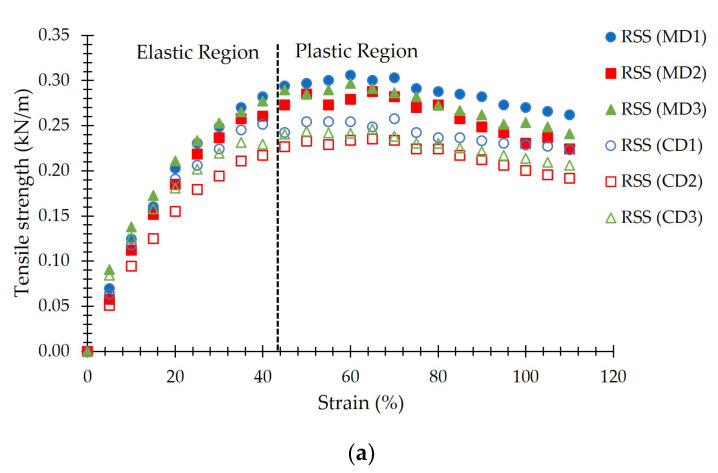

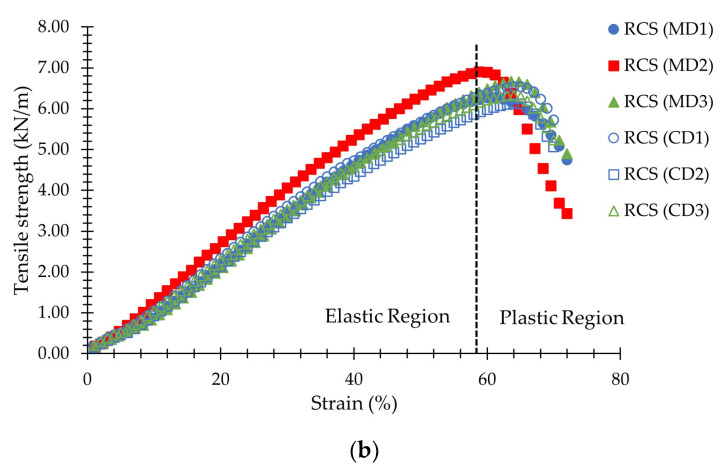
Tensile strength (**a**) RSS geogrid (**b**) RCS geogrid.

The reinforcement materials were geogrids made from para rubber, which consists of ribbed smoked sheet grade 3 (RSS) and rubber compound sheet. The RSS, which was called the RSS geogrid, was produced from coagulated latex sheets, which are smoked in an oven at a suitable temperature [22]. The thickness of RSS was between 2.50 mm and 3.90 mm [18]. According to ASTM D6637-01 [23], the ultimate tensile strength of the RSS geogrid was tested in the machine direction (MD) and the cross-machine direction (CD), which were repeated 3 times as shown in Figure 3a. The average ultimate tensile strength and tensile strength at 2% strain on the MD were approximately 0.298 kN/m and 0.067 kN/m, respectively. While those on the CD were approximately 0.246 kN/m and 0.075 kN/m, respectively.

To increase the ultimate tensile strength of the RSS geogrid, the RSS was improved into a rubber compound sheet (RCS) by adding the chemicals to raw rubber, which was RSS in this study. The chemicals components of RCS were RSS, zinc oxide (ZnO), stearic acid, rubber antioxidant (6PPD), CaCO_3_-HI-CAL CC, carbon black N330, silica, polyethylene glycol (PEG4000), paraffin oil, sulfur, and rubber accelerator (CBS) as shown in Table 1. The thickness of the RCS was between 2.50 mm to 3.50 mm. The ultimate tensile strength of the RCS geogrid [23] was tested in both MD and CD, which were repeated 3 times as shown in Figure 3b. The average ultimate tensile strength and tensile strength at 2% strain on the MD were approximately 6.614 kN/m and 0.251 kN/m, respectively. While those on the CD were approximately 6.327 kN/m and 0.269 kN/m, respectively. The ultimate tensile strength of the geogrid was typically varying from 0.25 to 14.4 kN/m [24].

**Figure 4:**
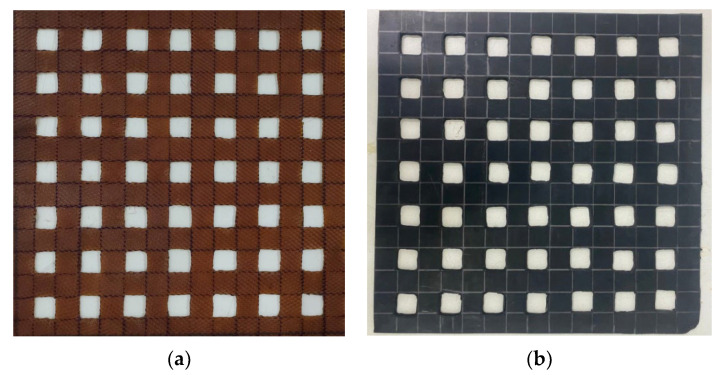
(**a**) RSS geogrid; (**b**) RCS geogrid.

Material factors affecting the interface shear strength coefficient of soil reinforcement consisted of the angle between the geogrid and shear direction, aperture size, aperture area, maximum tensile strength, and junction strength of the geogrid [9,10]. Therefore, the angle between the geogrid and shear direction, aperture size, and aperture area of the RSS geogrids and RCS geogrids were set as the control factors for standard comparison. The aperture area of RSS and RCS geogrids was 400 mm^2^, which was designed as a biaxial geogrid in the shape of a square measuring 20 mm × 20 mm and a spacing of 20 mm. The properties of the RSS and RCS geogrids, including tensile strength at 2% strain and ultimate tensile strength, are shown in Table 2. In addition, the RSS and RCS geogrids are shown in Figure 4.

## 3. Methodology

### 3.1. Number of Blows Per Compaction Layer

According to standard test methods for laboratory compaction characteristics of soil using modified effort (ASTM D1557) [25], the number of blows per compaction layer was determined. The height of the soil samples, which were compacted in the large-scale direct shear apparatus, was 100 mm (50 mm in the lower shear box and 50 mm in the upper shear boxes). The number of blows per compaction layer was calculated as follows [26,27]:

*E* = *NnWh*/*v* (1)

where:

*E* = compaction energy (kN-m/m^3^);
*N* = number of blows per layer (blow);
*n* = number of layers (layer);
*W* = hammer weight (kN);
*h* = height of drop (m);
*v* = volume of mold (m^3^).

**Figure 5:**
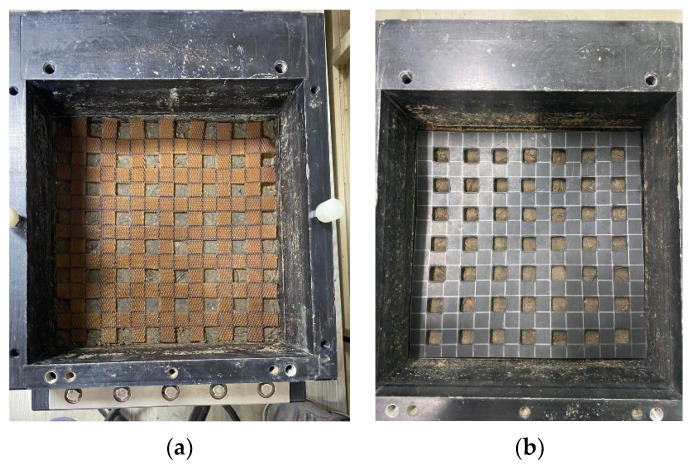
The RSS and RCS geogrids in the shear box: (**a**) RSS geogrid; (**b**) RCS geogrid.

From Equation 1, the compaction energy (*E*) was applied approximately 2700 kN-m/m^3^ to the compacted soil sample with 1212 blows. In this study, the compacted soil in the shear box was divided into 4 layers, which were 2 layers in each lower- and upper-shear boxes. Therefore, 303 blows were applied to each compaction layer. The RSS and RCS geogrids were placed at the interface of the lower- and upper-shear boxes as shown in Figure 5.

### 3.2. Optimum Moisture Content

**Figure 6:**
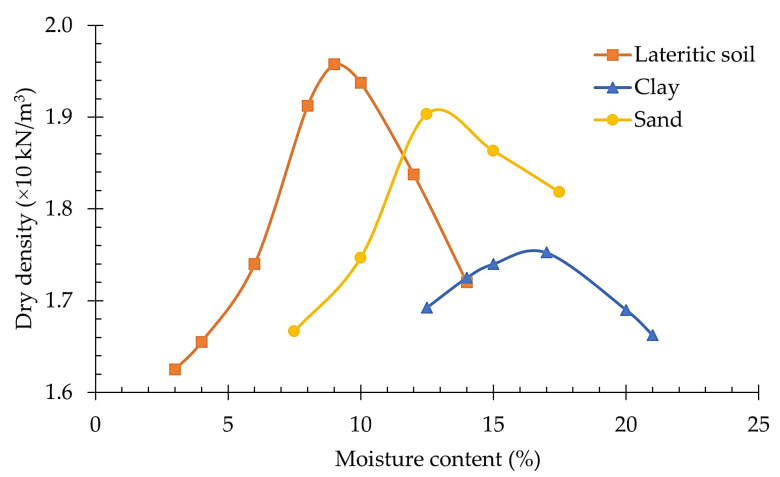
Compaction curve of sand, lateritic soil, and clay samples.

Moreover, the soil sample was compacted in the shear box with optimum moisture content at maximum dry density, which was determined by a modified compaction test (ASTM D1557) [25]. The compaction curve, which was the bell curve, showed the relationship between moisture content and dry density of soil samples as shown in Figure 6.

The peak of the compaction curve shows the optimum moisture content at maximum dry density. The optimum moisture content was 12.76%, 15.77%, and 8.80% for sand, clay, and lateritic soil, respectively. These values were referred to in order to prepare and control the density of soil samples during compaction in the shear boxes.

### 3.3. Large-Scale Direct Shear Test

The direct shear test is widely employed to determine the interaction mechanism, which is soil sliding, between soil and geogrid reinforcement [28]. Therefore, the large-scale direct shear test with the 20,000 lbf Large Direct Shear Machine (220–240 V/50 Hz) model 2012-HPF by Karol-Warner, Powell, OH, United States was applied in this study. The shear boxes consisted of upper- and lower-shear boxes with sizes 305 mm in length, 305 mm in width, and 50 mm in height.

**Figure 7:**
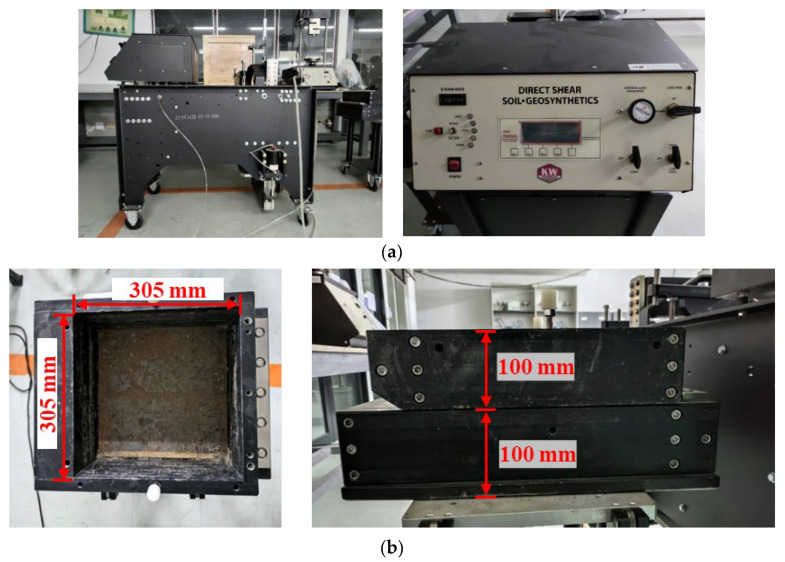
Large-scale direct shear apparatus: (**a**) large-scale direct shear apparatus; (**b**) shear box.

The horizontal force was applied to the lower shear box through the electrical jack that can control the displacement rate. The vertical stress was applied through a steel plate in a square shape with dimensions of 305 mm × 305 mm, which was plated on the top of the upper shear box. The steel plate was linked with an air-pressure pump to control the pressure, which was applied as vertical stress. The electrical sensors in the large-scale direct shear apparatus were used to measure and record real-time results, which were time, horizontal force, horizontal displacement, vertical pressure, and vertical displacement. The large-scale direct shear apparatus and the dimension of the shear box are shown in Figure 7a,b, respectively.

**Figure 8:**
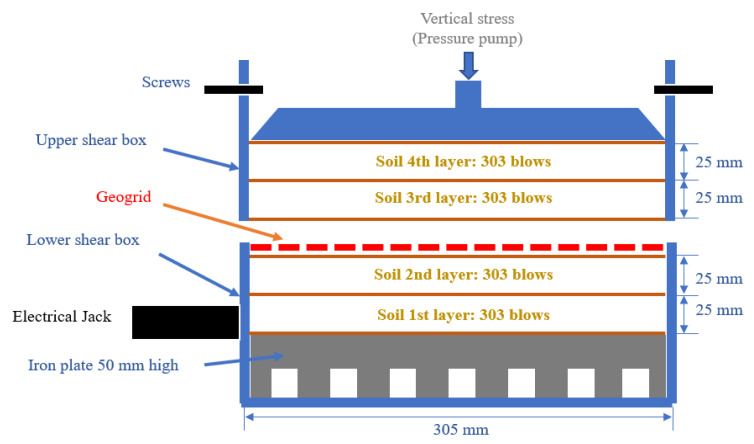
The position of the geogrid in the large-scale direct shear apparatus.

The shearing process was performed according to ASTM D5321 [29], and the normal stress of 30, 60, and 120 kN/m^2^ were applied to the soil samples in the shear box through the steel plate. The speed of horizontal displacement was fixed constantly at 1 mm/min. The upper-shear box was locked by screws to prevent the movement of the upper-shear box in both vertical and horizontal directions. Meanwhile, the lower-shear box was pushed by the electrical jack. The position of the geogrid in the shear box was shown in Figure 8.

The density of the compacted-soil sample in the box of large-scale direct shear was tested by the sand cone method for determining the relative compaction (RC). According to the requirements of the Thailand Department of Highways [30], the RC values must surpass 95%. As the results from the sand cone test, it was found that the RC values of all samples were over 95%, which were 95–96%, 97–100%, and 98–101% for sand, lateritic soil, and clay, respectively.

The testing results obtained from the large-scale direct shear test were normal stress, vertical displacement, horizontal force, and horizontal displacement. The shear stress was calculated from Equation 2 as follows:

*T* = *F/A*_c_ (2)

where:

*T* = shear stress (kN/m^2^);
*F* = shear force or horizontal force (kN);
*A*_c_ = corrected area (m^2^).

The area is the contracted area of the specimen, which decreases with the increase of sample displacement. Therefore, the corrected area must be calculated and applied to calculate the shear stress. The corrected area was calculated as in Equation 3:

*A_c_* = *A* − (*dH* × *W*) (3)

where:

*A*_c_ = corrected area (m^2^);
*A* = initial specimen contact area or area of shear box (m^2^);
*dH* = horizontal displacement (m);
*W* = width of shear box (m).

After the test, the relationship between shear stress and horizontal displacement, as well as between the shear stress and normal stress are determined. The ratio of the shear strength of the reinforced soil with the RSS geogrid or RCS geogrid and non-geogrid reinforcement (soil only) was recorded as the interface shear strength coefficient (*R*_in_) as follows [31]:

*R*_in_ = (σtan*δ* + *c*_i_)/(σ tan *ϕ* + *c*) (4)

where:

*σ* = normal stress;
δ = skin friction angle between soil and reinforcement material;
*c*_i_ = adhesion between soil and reinforcement material;
*ϕ* = internal friction angle between soil and soil;
*c* = cohesion between soil and soil.

## 4. Testing Conditions

The soil samples, which were used in this research, were sand, lateritic soil, and clay. All soil samples were tested under 3 conditions, which were non-geogrid reinforcement (soil only), reinforced soil by the RSS geogrid, and reinforced soil by the RCS geogrid, with the applied normal stress of 30, 60, and 120 kN/m^2^. All testing conditions were repeated 3 times for the accuracy of the results. Therefore, the testing conditions were divided into 27 conditions (Table 3) and names are as follows:

1. Sand UR refers to ordinary sand (sand only);

2. Sand RSS refers to RSS geogrid-reinforced sand backfill;

3. Sand RCS refers to RCS geogrid-reinforced sand backfill;

4. Lat UR refers to ordinary lateritic soil (lateritic soil only);

5. Lat RSS refers to RSS geogrid-reinforced lateritic soil backfill;

6. Lat RCS refers to RCS geogrid-reinforced lateritic soil backfill;

7. Clay UR refers to ordinary clay (clay only);

8. Clay RSS refers to RSS geogrid-reinforced clay backfill;

9. Clay RCS refers to RCS geogrid-reinforced clay backfill.

## 5. Results and Discussion

### 5.1. Shear Stress and Horizontal Displacement

**Figure 9:**
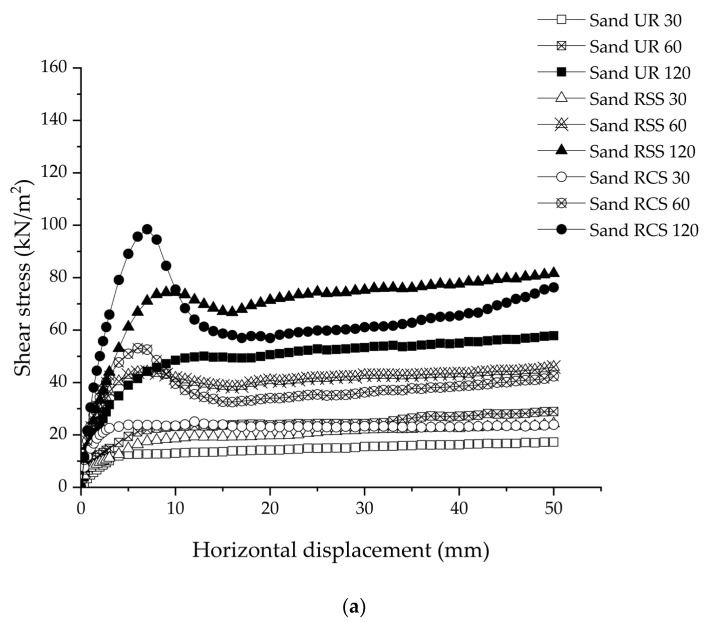

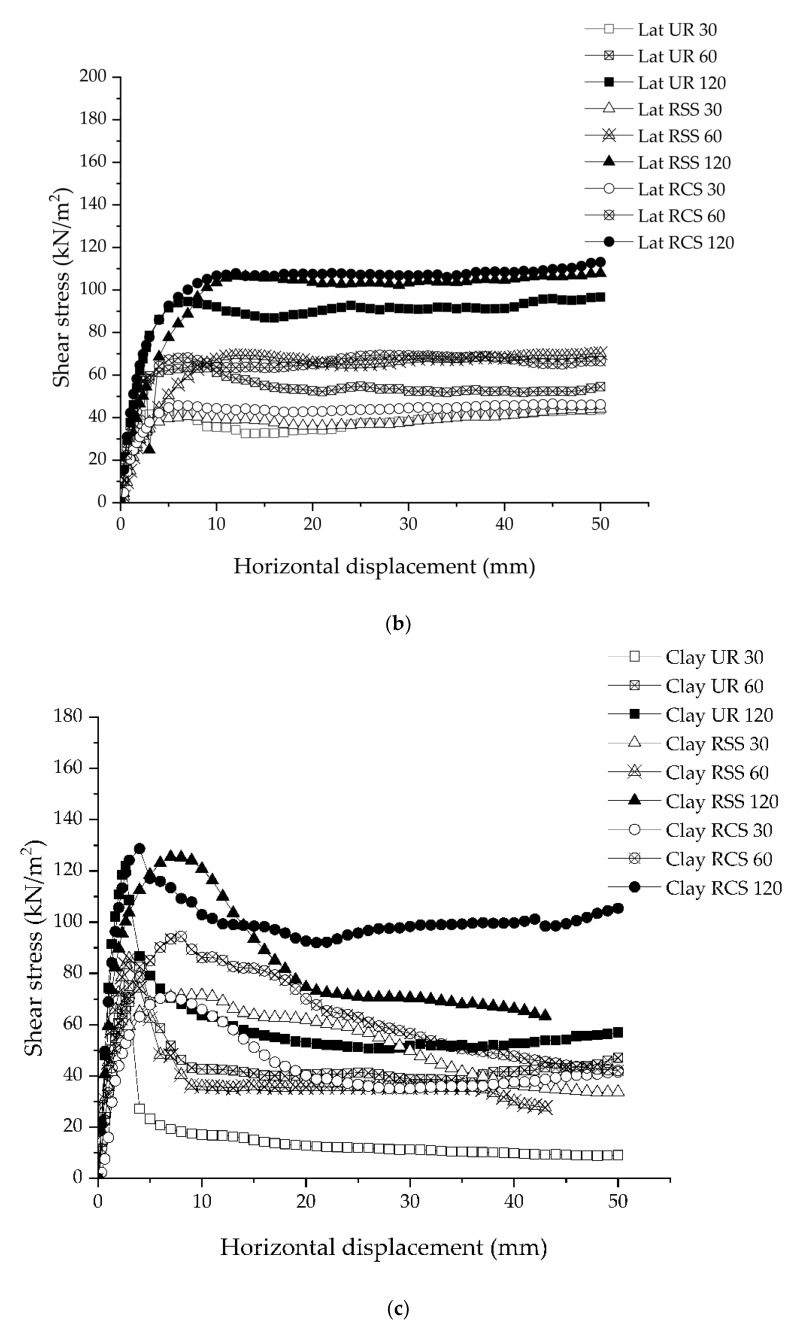
The relationship between shear stress and horizontal displacement of: (**a**) sand; (**b**) lateritic soil; (**c**) clay.

Figure 9 shows the relationship between the shear stress and horizontal displacement under the normal stress of 30, 60, and 120 kN/m^2^, respectively. The shear stress of sand increased rapidly until the maximum shear stress was achieved (Figure 9a). After reaching the peak, the shear stress decreased with an increase in the horizontal displacement. As a result, the rate of shear stress was linearly constant. The applied high-normal stress (120 kN/m^2^), the shear stress, and the horizontal displacement relationship presented the trend with shear stress as a bell shape, especially in the sand RCS sample. On the other hand, the peak points of shear stress or the bell shape were not observed in the sand UR samples and the sample under the normal stress of 30 kN/m^2^. The highest shear stress was 100.4 kN/m^2^ under the condition of Sand RCS 120. As a result, it was found that the occurrence of the bell shape graph in sand with RSS and sand with RCS presented the effect of RSS and RCS geogrids increasing the interlock between sand particles. This led to an increase in frictional resistance when the normal stress was applied to the sand sample.

Figure 9b shows the relationship between shear stress and horizontal displacement in lateritic soil. Shear stress increased rapidly in lateritic soil up to its maximum, and then it remained constant under all conditions as horizontal displacement increased. The highest shear stress occurred under the Lat RCS 120 condition, which was 115.4 kN/m^2^. Moreover, the shear stress of lateritic soil showed a similar trend to the shear stress of sand UR, which was the condition without geogrid reinforcement. Additionally, Sand RSS 30 and Sand RCS 30 both applied low-normal stress conditions. [32].

Considering all the clay samples, the shear stress of clay significantly increased after starting the shearing process until reaching the maximum shear stress (Figure 9c). Then shear stress decreased rapidly as a bell curve; this behavior agrees with the earlier result by Bergado (1993) [3], Gratchev and Sassa (2015) [33], and Dafalla (2012) [34]. The highest maximum shear stress of clay was obtained at 133.6 kN/m^2^ under Clay RCS 120.

Considering the same normal stress, the highest shear stress for all testing conditions was the RCS geogrid-reinforced soil backfill, followed by RSS geogrid-reinforced soil backfill, and ordinary soil, respectively. This was due to the mechanical properties of the RSS and RCS geogrids (Figure 3a,b). The elastic modulus, or Young’s modulus (*E*) is the relationship between tensile strength (σ) to strain (ε) in the linear elastic region, in which the material can return to original shape after removing the stress. The *E* is σ/ε, which was directly reflected in the shear behavior. Shear modulus (*G*) is the relationship of *E/*2 (1 + *υ*), where *υ* = Poisson’s ratio. Moreover, the shear stress (*τ*) is *G*/*γ*, where *γ* = shear strain.

According to these results, it can be concluded that the RCS geogrid has the highest tensile strength, which influenced an increase in shear stress of the RCS geogrid-reinforced soil backfill [35,36].

### 5.2. Shear Stress and Normal Stress

**Figure 10:**
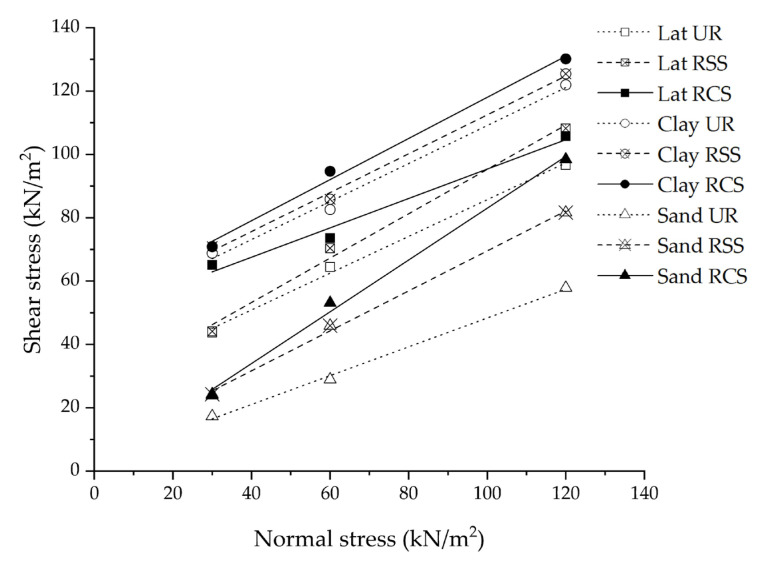
Mohr–Coulomb shear strength envelopes of sand, lateritic soil, and clay at all conditions.

Figure 10 shows the strength envelope, which is the relationship between shear stress (a peak of shear stress in Figure 9) and normal stress. The shear strength increased with an increase of normal stress for all testing conditions. The highest normal stress of all soil samples is approximately 120 kN/m^2^ since this value can prevent bond failure between soil and the geogrid [9].

The shear strength of clay was the highest followed by lateritic soil and sand, respectively. The highest shear strength of clay was due to the clay sample being compacted at the optimum moisture content (15.77%), which is lower than the plastic limit (39.74%). This means that the clay sample was prepared in a solid state. The clay with low moisture (lower than the plastic limit) is tough. The shear strength and compressive strength of clay in this state are significantly higher than in other states [37]. In addition, the minimum relative diameter (D_min_/D_50_) of lateritic soil was lower than that of sand. The decrease of D_min_/D_50_ has a remarkable effect on increasing the shear strength [38,39].

Moreover, there was a similar trend in the strength envelope for all soil samples, which were the highest at RCS geogrid reinforcement, followed by RSS geogrid reinforcement, and non-geogrid reinforcement, respectively. This is related to the research of Bergado et al. (1993) [3], which mentioned that the shear strength of soil and reinforcement material can exceed the soil itself since the shear resistance is mobilized by direct shear test. In addition, the tensile strength of the geogrid has an influence on reinforcement efficiency [9]. The tensile strength of the RCS geogrid was higher than that of the RSS geogrid. This caused higher reinforcement efficiency under the condition of RCS geogrid reinforcement.

The strength envelope of ordinary soil was used to determine the cohesion and internal friction angle between soil and soil, while the strength envelope of soil with geogrid-reinforcement material was used to determine the adhesion and skin friction between soil and the reinforcement material. The cohesion, internal friction angle, adhesion, and skin friction angle are shown in Table 4.

Table 4 shows that there is no obvious increasing trend in the strength development from cohesion to adhesion for all soil in this study, which is dependent on the interface between soil backfill and geogrid reinforcement. In the case of the sand, the adhesion between sand backfills and the RSS geogrid was 6.56 kN/m^2^, which was higher than the cohesion of ordinary sand (2.89 kN/m^2^). Whereas, the adhesion between sand backfills and the RCS geogrid was 1.27 kN/m^2^, which was lower than the cohesion of ordinary sand. The adhesion between lateritic soil backfills and the RCS geogrid (49.97 kN/m^2^) was higher than that of the RSS geogrid reinforcement (25.72 kN/m^2^), and cohesion of ordinary lateritic soil (28.19 kN/m^2^). With the clay-reinforced with RSS and RCS geogrids, the adhesion increased from 50.08 kN/m^2^ (ordinary clay) to 52.10 and 54.14 kN/m^2^, respectively.

The skin friction angles of the RSS geogrid-reinforced soil backfill (32.26° for sand, 35.01° for lateritic soil, and 31.55° for clay), and RCS geogrid-reinforced soil backfill (39.28° for sand, 36.19° for lateritic soil, and 33.01° for clay) were higher than the internal friction angle of all ordinary soil (24.48° for sand, 30.15° for lateritic soil, and 30.97° for clay). Considering all testing conditions, the most difference between internal friction angle and skin friction angle was found in the sand for the condition of Sand RCS, followed by Sand RSS, respectively.

Moreover, the skin friction angle of the RCS geogrid-reinforced soil backfill was the highest, followed by the RSS geogrid-reinforced soil backfill, and ordinary soil, respectively. As a result, it is evident that the interaction between soil backfill, and both the RSS and RCS geogrid reinforcements caused the variation in internal friction and cohesion after applying those RSS and RCS geogrid reinforcement materials [10].

### 5.3. Interface Shear Strength Coefficient (R_in_)

The interface shear strength coefficient is defined as *R*_in_. The *R*_in_ is the ratio between the shear strength of the soil with the geogrid reinforcement and ordinary soil. In the case where the *R*_in_ value is more than 1, this means that adding the geogrid reinforcement material can be used to improve the interface shear strength of ordinary soil. On the other hand, if the *R*_in_ value is less than 1, this means the occurrence of lower shear strength of the soil sample. The interaction mechanisms between soil and geogrid depend on the properties of reinforcement material and the strength of the soil [28].

From Table 5, the average *R*_in_ value of sand with geogrid reinforcement was the highest value among the three types of soil backfill samples, which were 1.479 and 1.654 for RSS geogrid reinforcement and RCS geogrid reinforcement, respectively. Those of lateritic soil with geogrid reinforcement were 1.075 and 1.233 for RSS geogrid reinforcement and RCS geogrid reinforcement, respectively. While the average *R*_in_ value of clay was the lowest average *R*_in_ values, which were 1.033 and 1.082 RSS geogrid reinforcement and RCS geogrid reinforcement, respectively.

From these results, it can be concluded that the RCS and RSS geogrids had the highest effects on sand, followed by lateritic soil, and clay, respectively. The geogrid reinforcement has more ability to improve the soil with a low shear strength [10,17] similar to the results of sand UR, which had the lowest shear strength and the highest *R*_in_. Moreover, the increasing interlock between soil particles with geogrid aperture led to the occurrence of the lateral restraint function [40], which was highly affected by the coarse-grained soil (sand and gravel) [41].

Comparing the *R*_in_ value under the same type of soil condition, the average *R*_in_ of the RCS geogrid reinforcement was significantly higher than those of the RSS geogrid condition, especially in sand samples. The tensile strength of the geogrid influences reinforcement efficiency [9,10].

## 6. Conclusions

For strengthening the soil samples, which included sand, lateritic soil, and clay, the RSS geogrid and RCS geogrid were suggested as reinforcement materials. A large-scale direct shear test was used to estimate the *R*_in_ to verify the effectiveness of geogrid reinforcement. The following conclusions could be drawn:

1. The RSS geogrid and RCS geogrid worked well to increase the soil’s shear strength.
2. The tensile strength of the RCS geogrid reinforcement made it superior to the RSS geogrid reinforcement for all types of soil.
3. In comparison to reinforced clay and lateritic soil with the RCS geogrid, the higher average *R*_in_ was clearly observed for reinforced sand. The sand was thus the best material for reinforcement by an RCS geogrid.

## Figures and Tables

**Table 1:** Composition of rubber compound sheet.

Chemicals Components	Amount (phr)
Ribbed smoked sheet grade 3 (RSS)	100
Zinc oxide (ZnO)	3
Stearic acid	1
Rubber antioxidant (6PPD)	1
CaCO_3_-HI-CAL CC	50
Carbon black N330	50
Silica	5
Polyethylene glycol (PEG4000)	1
Paraffin oil	30
Sulfur	2
Rubber accelerator (CBS)	1.5
Total phr	244.5

**Table 2:** Engineering properties of reinforcement material.

Properties	RSS Geogrid	RCS Geogrid
Aperture size (mm)	20 × 20	20 × 20
Aperture area (mm^2^)	400	400
Thickness (mm)	2.38	2.51
Ultimate tensile strength MD (kN/m)	0.298	6.614
Ultimate tensile strength CD (kN/m)	0.246	6.327
Tensile strength at 2% strain MD (kN/m)	0.067	0.251
Tensile strength at 2% strain CD (kN/m)	0.075	0.269

**Table 3:** Testing conditions.

Soil Types	Reinforcement Material	Normal Stress (kN/m^2^)		
		30	60	120
Sand	Non-geogrid reinforced	Sand UR 30	Sand UR 60	Sand UR 120
	RSS geogrid	Sand RSS 30	Sand RSS 60	Sand RSS 120
	RCS geogrid	Sand RCS 30	Sand RCS 60	Sand RCS 120
Lateritic soil	Non-geogrid reinforced	Lat UR 30	Lat UR 60	Lat UR 120
	RSS geogrid	Lat RSS 30	Lat RSS 60	Lat RSS 120
	RCS geogrid	Lat RCS 30	Lat RCS 60	Lat RCS 120
Clay	Non-geogrid reinforced	Clay UR 30	Clay UR 60	Clay UR 120
	RSS geogrid	Clay RSS 30	Clay RSS 60	Clay RSS 120
	RCS geogrid	Clay RCS 30	Clay RCS 60	Clay RCS 120

**Table 4:** Cohesion, internal friction angle, adhesion, and skin friction angle for all soil samples.

Testing Conditions	Cohesion (kN/m^2^)	Adhesion (kN/m^2^)	Internal Friction Angle (Degree)	Skin Friction Angle (Degree)
Sand UR	2.89	-	24.48	-
Sand RSS	-	6.56	-	32.26
Sand RCS	-	1.27	-	39.28
Lat UR	28.19	-	30.15	-
Lat RSS	-	25.72	-	35.01
Lat RCS	-	49.97	-	36.19
Clay UR	50.08	-	30.97	-
Clay RSS	-	52.10	-	31.55
Clay RCS	-	54.14	-	33.01

**Table 5:** The average *R*_in_ for all testing samples.

Samples	*R*_in_			
	Normal Stress = 30	Normal Stress = 60	Normal Stress = 120	Average
Sand UR	1	1	1	1
Sand RSS	1.538	1.469	1.430	1.479
Sand RCS	1.563	1.669	1.730	1.654
Lat UR	1	1	1	1
Lat RSS	1.026	1.076	1.123	1.075
Lat RCS	1.396	1.229	1.075	1.233
Clay UR	1	1	1	1
Clay RSS	1.036	1.033	1.030	1.033
Clay RCS	1.081	1.082	1.082	1.082

## Data Availability

The data presented in this study are available on request from the corresponding author.

## References

[1] Kiersnowska A., Fabianowski W., Koda E. (2020). The Influence of the Accelerated Aging Conditions on the Properties of Polyolefin Geogrids Used for Landfill Slope Reinforcement. Polymers.

[2] Yousif H.F. (2015). Effect of Expansive Subgrade Soil on Reinforced Subbase Layer. Master’s Thesis.

[3] Bergado D.T., Chai J.C., Abiera H.O., Alfaro M.C., Balasubramaniam A.S. (1993). Interaction between cohesive-frictional soil and various grid reinforcements. Geotext. Geomembr..

[4] Nagrale P., Sawant P., Pusadkar S. Laboratory investigations of reinforced subgrade soils. Proceedings of the Indian Geotechnical Conference.

[5] Hufenus R., Rueegger R., Banjac R., Mayor P., Springman S.M., Brönnimann R. (2006). Full-scale field tests on geosynthetic reinforced unpaved roads on soft subgrade. Geotext. Geomembr..

[6] Cai X., Feng J., Li S., Xu H., Liu W., Huang X. (2022). Study on Interface Interaction between Uniaxial Geogrid Reinforcement and Soil Based on Tensile and Pull-Out Tests. Sustainability.

[7] Bai Q., Liu J., Wang Y., Du H., Wang B. (2022). Experimental Investigation of Interface Characteristics between Geogrid and Coarse-Grained Soil in a Seasonally Frozen Area. Appl. Sci..

[8] Raja M.N.A., Shukla S.K. (2020). Ultimate bearing capacity of strip footing resting on soil bed strengthened by wraparound geosynthetic reinforcement technique. Geotext. Geomembr..

[9] Palmeira E.M., Milligan G.W.E. (1989). Large scale direct shear tests on reinforced soil. Soils Found..

[10] Sakleshpur V.A., Prezzi M., Salgado R., Siddiki N.Z., Choi Y.S. (2019). Large-scale direct shear testing of geogrid reinforced aggregate base over weak subgrade. Int. J. Pavement Eng..

[11] Artidteang S., Bergado D.T., Chaiyaput S., Tanchaisawat T., Lam L.G. (2016). Performance of ruzi grass combined with woven limited life geotextiles (LLGS) for soil erosion control. J. Lowl. Technol. Int..

[12] Artidteang S., Tanchaisawat T., Bergado D.T., Chaiyaput S. (2015). Natural fibers in reinforcement and erosion control applications with limited life geosynthetics. Ground Improv. Case Hist. Compact. Grouting Geosynth..

[13] Artidteang S., Bergado D.T., Chaiyaput S., Tanchaisawat T. (2015). Embankment reinforced with limited life geotextiles on soft clay. Proc. Inst. Civ. Eng. Ground Improv..

[14] Chaiyaput S., Bergado D.T., Artidteang S. (2014). Measured and simulated results of a kenaf limited life geosynthetics (LLGs) reinforced test embankment on soft clay. Geotext. Geomembr..

[15] Sarsby R. (2007). Limited-life geosynthetics. Geosynthetics in Civil Engineering.

[16] Mwasha A. (2009). Designing bio-based geotextiles for reinforcing an embankment erected on soft soil. Mater. Des..

[17] Arwaedo N., Chaiyaput S., Sukchaisit O., Auephattayakorn K., Deawtipsukon S. Effect of ribbed smoked sheet on CBR strength of lateritic soil. In Proceeding of the 5th International Conference on Smart Materials and Nanotechnology.

[18] Chaiyaput S., Arwaedo N., Jamsawang P., Ayawanna J. (2018). Natural para rubber in road embankment stabilization. Appl. Sci..

[19] Brydson J.A. (1995). Thermoplastic elastomers: Properties and applications. Rapra Rev. Rep..

[20] (2020). Standard Practice for Classification of Soils for Engineering Purposes (Unified Soil Classification System).

[21] (2018). Standard Test Methods for Liquid Limit, Plastic Limit, and Plasticity Index of Soils.

[22] (2022). Rubber Authority of Thailand, Ribbed Smoked Sheet. https://km.raot.co.th/km-knowledge/detail/198/.

[23] (2001). Standard Test Method for Determining Tensile Properties of Geogrids by the Single or Multi-Rib Tensile Method.

[24] Al-Omari R.R., Fekheraldin M.K. Measurement of tensile properties of geogrids. Proceedings of the 2nd International Conference on Geotechnique, Construction Materials and Environment.

[25] (2021). Standard Test Methods for Laboratory Compaction Characteristics of Soil Using Modified Effort (56,000 ft-lbf/ft3 (2700 kN-m/m3)).

[26] Das B.M. (2013). Principles of Geotechnical Engineering.

[27] Civil Engineering Notes, Standard Proctor Test. http://civilengineering-notes.weebly.com/compaction-test---proctor-test.html.

[28] Jewell R.A., Milligan G.W.E., Sarsby R.W., Dubois D. Interaction between soil and geogrid. Proceedings of the Polymer Grid Reinforcement.

[29] (2021). Standard Test Method for Determining the Shear Strength of Soil-Geosynthetic and Geosynthetic-Geosynthetic Interfaces by Direct Shear.

[30] (1989). Standard Drawing for Highway Design and Construction.

[31] Bergado D.T., Chai J.C. (1994). Pullout force/displacement relationship of extensible grid reinforcements. Geotext. Geomembr..

[32] Liu C.N., Ho Y.H., Huang J.W. (2009). Large scale direct shear tests of soil/PET-yarn geogrid interfaces. Geotext. Geomembr..

[33] Gratchev I., Sassa K. (2015). Shear strength of clay at different shear rates. J. Geotech. Geoenviron. Eng..

[34] Dafalla M.A. (2013). Effects of clay and moisture content on direct shear tests for clay-sand mixtures. Adv. Mater. Sci. Eng..

[35] Kamalzare M., Moayed R.Z. (2011). Influence of geosynthetic reinforcement on the shear strength characteristics of two-layer sub-grade. Acta Geotech. Slov..

[36] Umashankar B., Chennarapu H., Mouli S.S. (2015). Interface Properties of Metal-Grid and Geogrid Reinforcements with Sand. IFCEE, Proceedings of the International Foundations Congress and Equipment Expo 2015, San Antonio, TX, USA, 17–21 March 2015.

[37] Malizia J.P., Shakoor A. (2018). Effect of water content and density on strength and deformation behavior of clay soils. Eng. Geol..

[38] Hazout L., Cherif T.A., Mahmoudi Y., Belkhatir M. (2022). Deformation characteristics of natural river sand under compression loading incorporating extreme particle diameters impacts. Mar. Georesources Geotechnol..

[39] Hazout L., El-Abidine Z., Belkhatir M., Schanz T. (2017). Evaluation of static liquefaction characteristics of saturated loose sand through the mean grain size and extreme grain sizes. Geotech. Geol. Eng..

[40] Cuelho E., Perkins S., Morris Z. (2014). Relative Operational Performance of Geosynthetics Used as Subgrade Stabilization.

[41] Zhao Y., Yang G., Wang Z., Yuan S. (2022). Research on the effect of particle size on the interface friction between geogrid reinforcement and soil. Sustainability.

